# Excessive dietary intake of vitamin A reduces skull bone thickness in mice

**DOI:** 10.1371/journal.pone.0176217

**Published:** 2017-04-20

**Authors:** Thomas Lind, Caroline Öhman, Gabriela Calounova, Annica Rasmusson, Göran Andersson, Gunnar Pejler, Håkan Melhus

**Affiliations:** 1 Department of Medical Sciences, Uppsala University, Uppsala, Sweden; 2 Department of Engineering Sciences, Division of Applied Materials Science, The Ångström Laboratory, Uppsala University, Uppsala, Sweden; 3 Department of Laboratory Medicine, Division of Pathology, Karolinska University Hospital Huddinge, Karolinska Institute, Stockholm, Sweden; 4 Department of Medical Biochemistry and Microbiology, Uppsala University, Uppsala, Sweden; 5 Department of Biomedical Science and Veterinary Public Health, Swedish University of Agricultural Sciences, Uppsala, Sweden; University of Texas Southwestern Medical Center, UNITED STATES

## Abstract

Calvarial thinning and skull bone defects have been reported in infants with hypervitaminosis A. These findings have also been described in humans, mice and zebrafish with loss-of-function mutations in the enzyme CYP26B1 that degrades retinoic acid (RA), the active metabolite of vitamin A, indicating that these effects are indeed caused by too high levels of vitamin A and that evolutionary conserved mechanisms are involved. To explore these mechanisms, we have fed young mice excessive doses of vitamin A for one week and then analyzed the skull bones using micro computed tomography, histomorphometry, histology and immunohistochemistry. In addition, we have examined the effect of RA on gene expression in osteoblasts *in vitro*. Compared to a standard diet, a high dietary intake of vitamin A resulted in a rapid and significant reduction in calvarial bone density and suture diastasis. The bone formation rate was almost halved. There was also increased staining of tartrate resistant acid phosphatase in osteocytes and an increased perilacunar matrix area, indicating osteocytic osteolysis. Consistent with this, RA induced genes associated with bone degradation in osteoblasts *in vitro*. Moreover, and in contrast to other known bone resorption stimulators, vitamin A induced osteoclastic bone resorption on the endocranial surfaces.

## Introduction

The skull bones protect the brain from traumatic injury. In spite of this important function, studies on determinants of skull bone thickness are very few. Skull bones (calvaria) are formed via direct ossification of connective tissue (intramembranous ossification), which is in contrast to long bones that use a cartilage template (endochondral ossification).

Vitamin A (retinol) is an essential micronutrient, which only can be derived from the diet as no animal species has the capability for de novo synthesis [1,2]. Vitamin A is also the only known molecule to induce spontaneous fractures in animals [1] and in humans an excessive vitamin A intake has been associated with an increased risk of hip fracture [3–5]. Studies in rodents have shown that a high intake of vitamin A leads to a thinning of the long bones due to both increased periosteal bone resorption and reduced bone formation [6]. Moreover, rats with hypervitaminosis A develop perforations in the scapula and mandible [7], suggesting that also flat bones can be affected. It is well established that vitamin A and other retinoids induce bone resorption in calvaria cultured *in vitro* [8–11], but whether excessive dietary intake of vitamin A has deleterious effects on the calvaria in rodents is unknown.

Case reports indicate that hypervitaminosis A can affect the calvaria in humans. Thin skull bones [12] and skull bone lesions [13] in infants were reported already in the early 1950s, and a decade later Woodard et al. [14] described bone destruction and delayed ossification of the parietal bone. Of special interest is that calvarial thinning was described in 1965 in five Swedish infants with vitamin A intoxication during their first half-year of life [15]. The periods of overdosing were short (1–3 months) and the doses considerably lower in comparison with those of cases reported earlier. During the 1950s, AD-vitamins replaced cod liver oil as the prophylaxis against rickets given to all infants and children in Sweden. After 1955, AD-vitamins soluble in water were introduced and increasingly used. Although it was known that an aqueous dispersion of vitamin A gave about four times higher blood concentration, the vitamin A content had remained the same, corresponding to a daily dose of about 2.5–3.0 mg [16]. All five infants had received water-soluble AD-preparations. Radiography showed calvarial thinning [15]. (The vitamin A content in AD-vitamins in Sweden was therefore later reduced, and removed completely in 2009).

Vitamin A is taken up from the blood stream by target cells and is converted to its active metabolite, retinoic acid (RA), inside the cell. CYP26B1, a member of the family of oxidizing P450 enzymes (CYP26 A, B, C), specifically inactivates RA inside the cell. Humans, mice and zebrafish lacking a functional CYP26B1 gene all have calvarial defects and large perforations in the parietal bones [17,18], indicating that these effects are indeed caused by excessive levels of vitamin A and that evolutionary conserved mechanisms are involved.

To investigate these mechanisms we have fed young mice amounts of vitamin A known to result in hypervitaminosis A and then analyzed the calvarial bones using micro computed tomography (μCT), histomorphometry, histology, immunohistochemistry and MC3T3-E1 cell culture experiments.

## Results

### Vitamin A reduces calvarial thickness and bone formation

**Fig 1:**
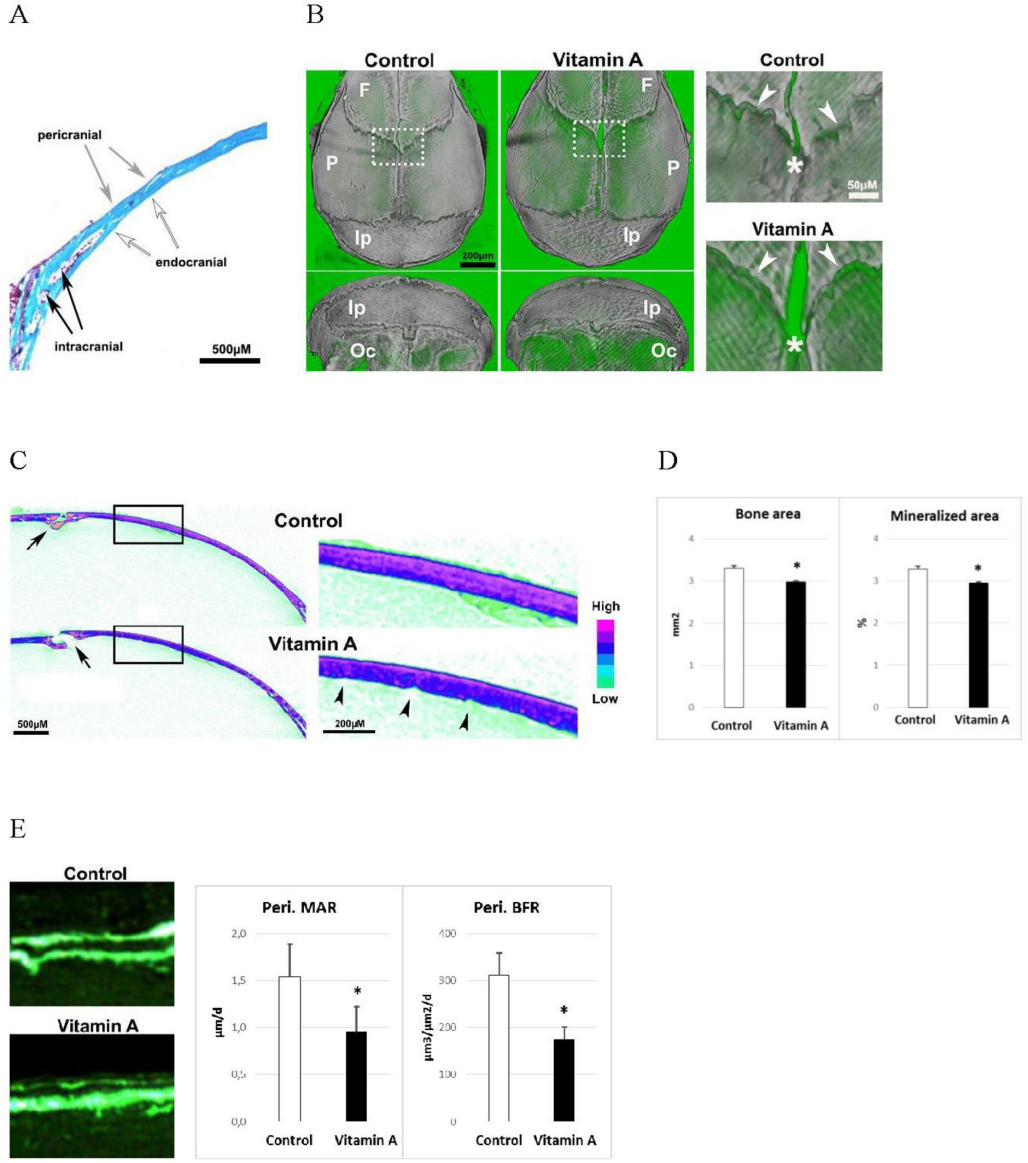
The calvarial bone and osteoblast phenotype. **A)** Illustration of peri- (peri), endo- (endo) and intra- (intra) cranial bone surfaces. **B)** Left panel: μCT picture of a pericranial and a dorsal view of isolated skull bones from mice fed excessive doses of vitamin A and control mice. F = frontal, P = parietal, Ip = Interparietal and Oc = occipital. Right panel are high power pictures of boxed areas in left panel, and show the intersection of the coronal (arrowheads) and sagittal (asterisk) sutures. **C)** Left panel: representative μCT pictures of transverse sections at the mid parietal bone (sagittal suture, arrow). Right panel: high power pictures of boxed area in the left panel. Arrowheads highlight the rougher endocranial surface in calvarial bone from vitamin A mice. **D)** Histomorphometric analyses of transverse bone area and mineralized area (n = 4/group). **E)** Left panel: pictures showing calcein double labelling of pericranial surface. Right panel: dynamic histomorphometric results, from analysis of the calcein double labeling. Pericranial mineral apposition rate (Peri. MAR) and pericranial bone formation rate (Peri. BFR) (n = 4/group). Results are given as means + SD. * p < 0.05.

As in our previous study in rats [19] with one week of standard diet supplemented with 1700 IU vitamin A/g pellet, the mice showed classic symptoms of hypervitaminosis A with a 10% reduced body weight (p<0.05) and a 13% reduced cross-sectional area (p<0.001) of the femur (measured by peripheral quantitative computed tomography, pQCT) while its length was not significantly affected (-1.1%, p = 0.08) compared to controls. Fig 1A describes the different calvarial bone surfaces analyzed in this study. High resolution μCT images showed that the parietal, frontal and occipital skull bones from mice fed excess vitamin A were less dense, whereas the interparietal bone was less affected (Fig 1B). High power images showed suture diastasis along the frontal/sagittal sutures whereas the coronal suture appeared less affected. Transverse views of μCT pictures displayed in Fig 1B showed, again, frontal/sagittal suture diastasis and bone hypoplasia in hypervitaminosis A animals (Fig 1C). High power versions of these pictures further revealed reduced bone density on the endocranial side together with increased bone surface roughness (Fig 1C). Histomorphometric analysis showed that vitamin A reduced both the bone area and mineralized area by 10% (Fig 1D). In addition, calcein double labeling experiments demonstrated a diminished osteoblast function on the pericranial side of the parietal bone with a reduced mineral apposition (-38%) and bone formation rate (-44%) (Fig 1E). The endo- and intracranial bone and mineral apposition rates were not affected by hypervitaminosis A (S1 Fig).

### Vitamin A increases bone resorption on the endocranial surfaces

**Fig 2:**
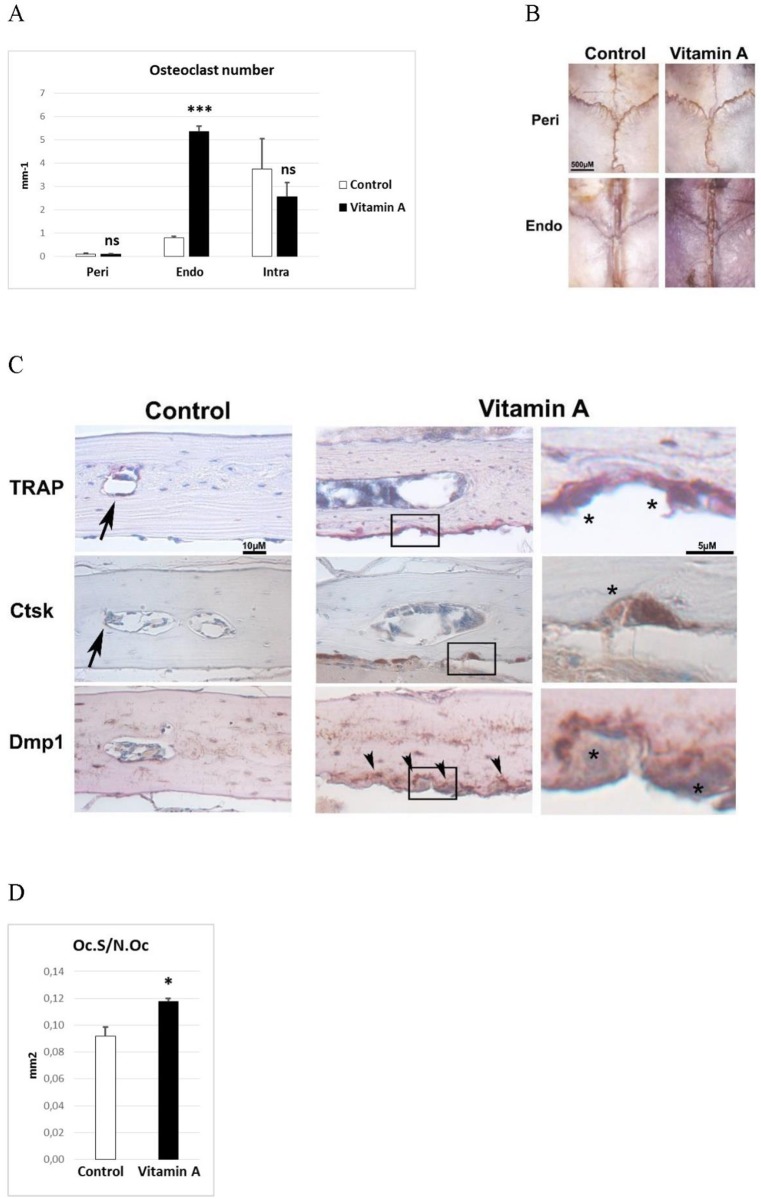
The osteoclast phenotype. **A)** Osteoclast number from histomorphometric analysis of pericranial (Peri), endocranial (Endo) and intracranial (Intra) surfaces (n = 4/group). **B)** Tartrate resistant acid phosphatase (TRAP) stained (red) intact calvaria. Photograph from pericranial (Peri) and endocranial (Endo) side. **C)** Representative pictures of TRAP staining (red) and immunohistochemical staining (brown) for cathepsin K (Ctsk) and dentin matrix protein 1 (Dmp1) of decalcified calvaria sections. Arrows indicate small, flat osteoclasts (TRAP and Ctsk) present in control tissue. Arrowheads indicate Dmp1 staining close to the endocranial osteoclasts, only found in bone from vitamin A animals. Right panel under “Vitamin A” shows high power pictures of boxed areas where asterisks indicate large osteoclasts only found in vitamin A animals. **D)** Osteoclast size determined as the osteoclast surface (Oc.S) divided by the osteoclast number (N.Oc) (n = 4/group). Results are given as means + SD. ns = not statistically significant, * p < 0.05 and *** p < 0.001.

As vitamin A is a well-known inducer of osteoclast activity, we counted osteoclasts on the peri-, intra- and endocranial bone surfaces using histomorphometry. In controls, most of the osteoclasts were intracranial with approximately a 4-fold lower number on the endocranial surface (Fig 2A). The pericranial surface presented only few osteoclasts. In calvaria of animals fed excess vitamin A there was a >6-fold increase in osteoclast number on the endocranial surface (Fig 2A). The intracranial surface showed a tendency to reduced number, although not statistically significant. The few pericranial osteoclasts were not affected by vitamin A intake. The conspicuous increase of endocranial osteoclasts was even visible by eye in intact calvaria stained for tartrate-resistant acid phosphatase (TRAP) activity, an enzyme highly expressed in osteoclasts (Fig 2B). In line with this, staining of decalcified paraffin-sections for the osteoclast markers cathepsin K (Ctsk) and TRAP showed that in controls most of the staining was found intracranially (Fig 2C). In contrast, most of the osteoclast staining was on the endocranial bone surface in animals fed excessive amounts of vitamin A. These osteoclasts appeared very active as they were voluminous and closely attached to the bone surface in distinct resorption pits. Furthermore, as osteocytes have been suggested to be the main producers of the key osteoclast inducer/activator, tumor necrosis factor (ligand) superfamily, member 11 (Tnfsf11, RANKL), efforts were made to detect this scarcely expressed protein using immunohistochemistry, but without success. Instead, we could show that osteocytes in contact with the endocranial osteoclasts in hypervitaminosis A animals displayed increased staining intensity for the osteocyte marker Dmp1, compared to endocranial osteocytes in control mice (Fig 2C). Osteoclast size, as determined by its bone surface contact length, was increased by 28% in hypervitaminosis A animals compared to controls (Fig 2D).

### Vitamin A increases Icam1 staining of the dura mater membrane covering the endocranial bone surface

**Fig 3:**
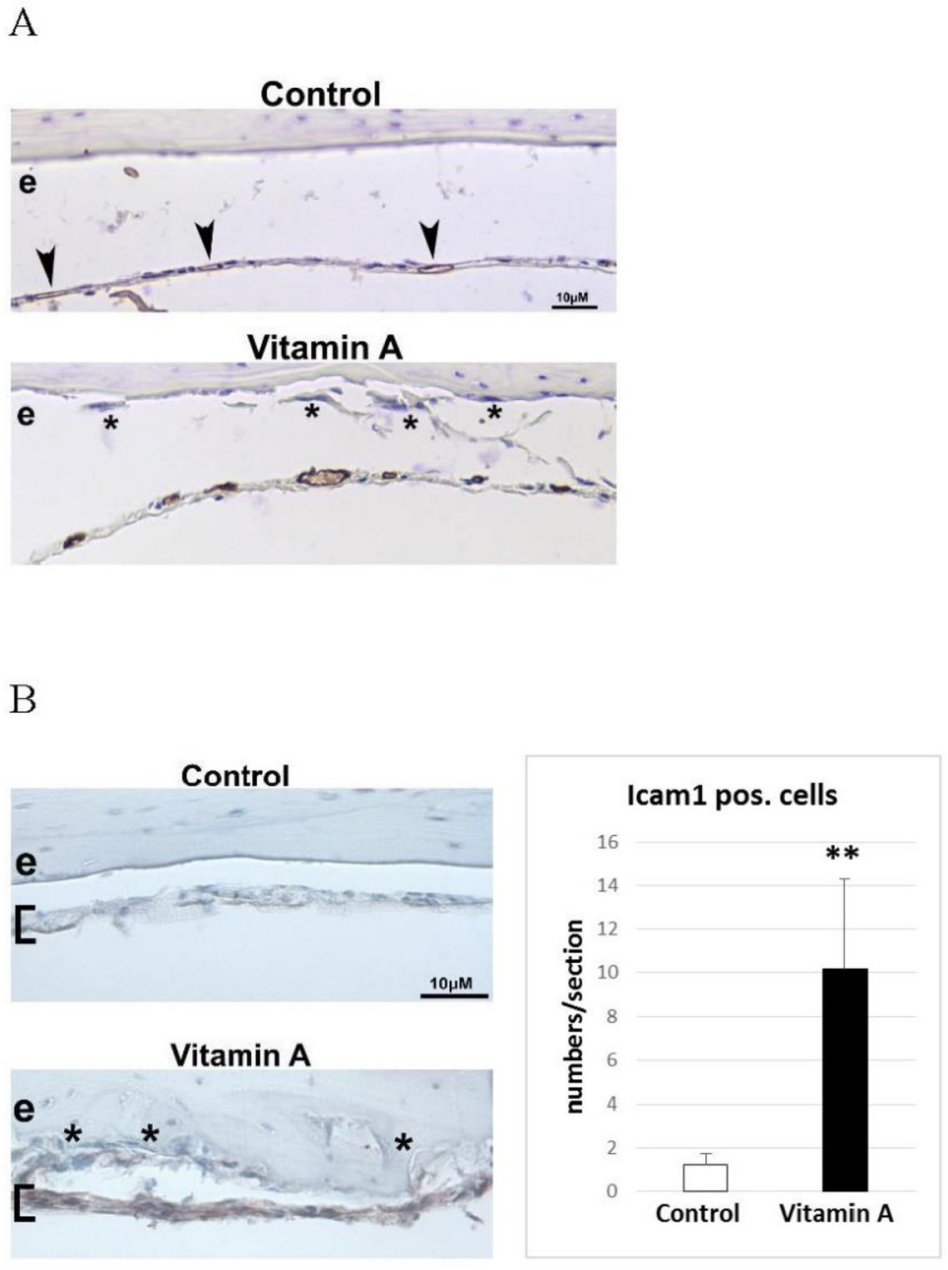
Endocranial associated dura mater blood vessels and Icam1 positive cells. **A)** Representative immunohistochemical staining (brown) for platelet/endothelial cell adhesion molecule 1 (Pecam1) positive blood vessels in decalcified calvaria sections. Arrowheads indicate small and flat Pecam1 positive blood vessel present in the dura mater membrane (covering the endocranial bone surface) of control tissue. In the “Vitamin A” panel the Pecam1 positive blood vessels are readily visible and appear engorged. Asterisks indicate osteoclasts in close proximity to the enlarged vessels, a combination only found in vitamin A animals. e = endocranial bone surface **B)** Immunohistochemical staining (brown) for intercellular adhesion molecule 1 (Icam1) in decalcified calvaria sections. Brackets indicate position of the dura mater membrane which is highly Icam1 positive only in vitamin A animals. Asterisks indicate osteoclasts in close proximity to the Icam1 positive cells, only found in vitamin A animals. Right panel shows the number of Icam1 positive cells/section in the dura mater (n = 4 and 6 sections/group and each group contain sections from 3 different individuals). Results are given as means + SD. ** p < 0.01.

As osteoclast precursors are recruited from the blood stream we next stained for presence of endothelial cells around calvarial bone with platelet/endothelial cell adhesion molecule 1 (Pecam1). In controls, small and flat Pecam1 positive blood vessel cells could be found in the dura mater membrane covering the endocranial bone surface (Fig 3A). Notably, the Pecam1 positive blood vessel cells in the dura mater from mice fed excess vitamin A appeared engorged and close to endocranial osteoclasts. Next, staining for Icam1, a key endothelial molecule involved in active recruitment and transendothelial migration of preosteoclasts from the blood, revealed increased staining in hypervitaminosis A animals (Fig 3B). These Icam1 positive cells were abundant just beneath the osteoclast-rich endocranial bone surface (Fig 3B). Quantification showed an approximately 5-fold increase of Icam1- positive cells in hypervitaminosis A compared to controls (Fig 3B). No apparent differences were observed for the blood vessels found close to the intra- or pericranial bone surfaces.

### Vitamin A induces osteocytic osteolysis

**Fig 4:**
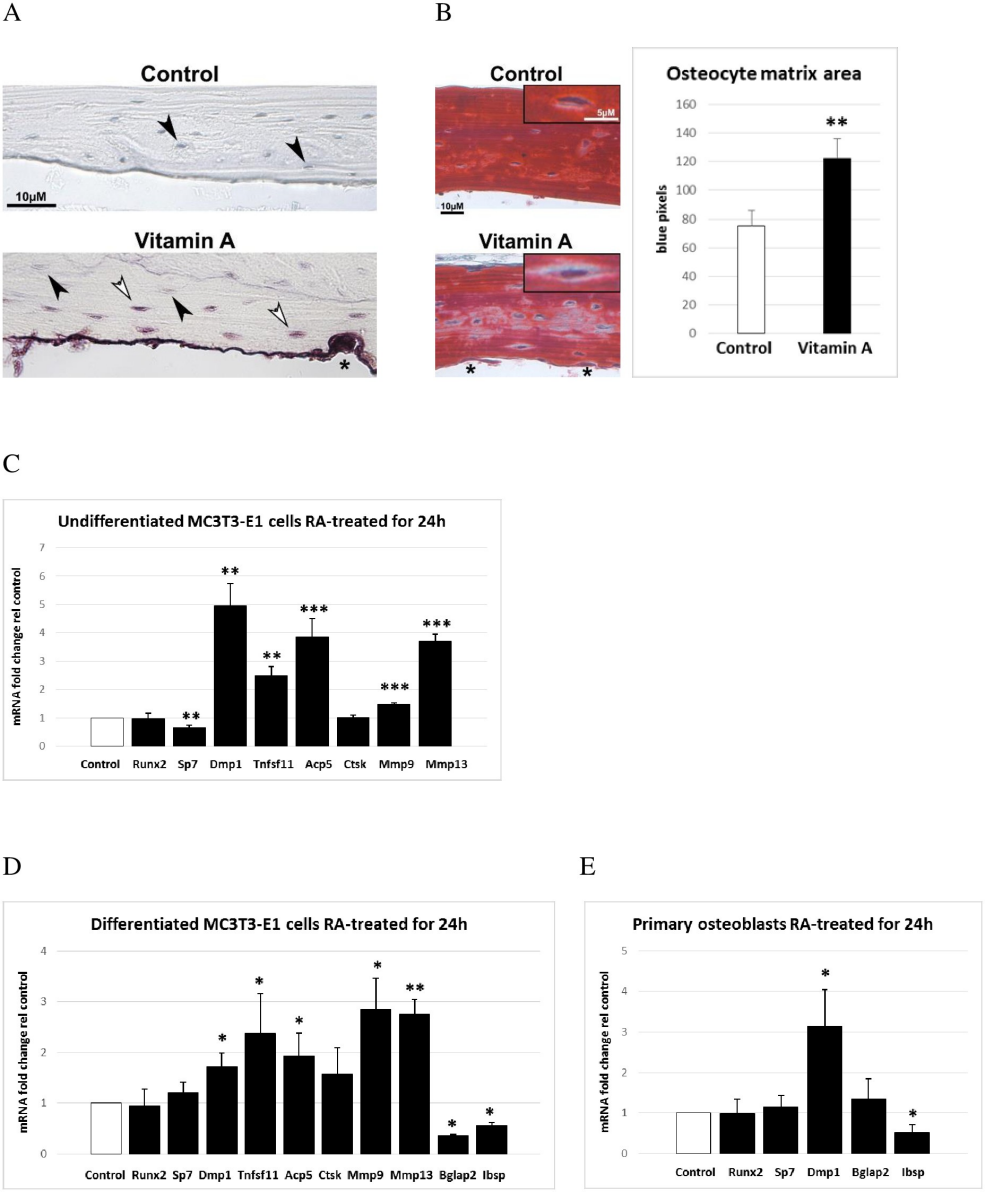
Osteocyte phenotype and RA-induced gene expression in osteoblastic cells. **A)** Representative picture of TRAP-stained decalcified bone section from mice fed excessive doses of vitamin A and controls. White arrowheads show TRAP-positive osteocytes close to the osteoclast rich endocranial surface and black arrowheads show TRAP-negative osteocytes. The asterisk indicates osteoclast attached to bone. **B)** Left panel shows a representative picture of Trichrome stained decalcified bone sections. Inset black box shows magnified osteocytes used for measurement of perilacunar matrix area (blue stain). The asterisks indicate osteoclasts. The right panel shows osteocyte perilacunar matrix area measured as number of blue pixels, analyzed from pictures of Trichrome stained bone sections (n = 10 osteocytes/group and each group contain sections from 3 different individuals). **C)** Osteoblastic (MC3T3-E1) gene expression after 24h of 400 nM retinoic acid (RA) treatment as determined by quantitative RT-PCR analysis. Runx2 (runt related transcription factor 2), Sp7 (Sp7 transcription factor 7, osterix), Dmp1 (dentin matrix protein 1), Tnfsf11 (tumor necrosis factor ligand superfamily member 11, RANKL), Acp5 (acid phosphatase 5, tartrate resistant, TRAP), Ctsk (cathepsin K), Mmp9 (matrix metalloproteinase 9) and Mmp13 (matrix metalloproteinase 13). **D)** Mature osteoblastic/osteocytic (differentiated MC3T3-E1 cells) gene expression after 24h of 400 nM retinoic acid (RA) treatment as determined by quantitative RT-PCR analysis. Bglap2 (bone gamma-carboxyglutamate protein 2, osteocalcin), Ibsp (integrin binding sialoprotein) and as described in (C). **E)** Primary mouse calvaria osteoblast gene expression after 24h of 400 nM retinoic acid (RA) treatment as determined by quantitative RT-PCR analysis. Results are given as means + SD. * p < 0.05, ** p < 0.01 and *** p < 0.001.

Close examination of the TRAP-stained bone sections showed that in addition to the intensely TRAP-positive osteoclasts on the endocranial surface, some osteocytes were also positive for TRAP (Fig 4A). To investigate changes in the connective tissue surrounding the osteocytes we stained decalcified bone sections with Trichrome, which stains bone red and cartilage blue. This revealed that hypervitaminosis A was associated with an increase in blue staining of the perilacunar space around osteocytes (Fig 4B). Measurement of the non-bone matrix amount (as blue pixels) showed that vitamin A increased this area around osteocytes with approximately 60% (Fig 4B). Finally, and in agreement with this being a direct effect of vitamin A, we showed that treatment of osteoblastic cells *in vitro* with retinoic acid (RA), i.e. the active metabolite of vitamin A, rapidly increased not only Acp5 (TRAP) mRNA expression but also expression of two collagenases, Mmp9 and Mmp13 (Fig 4C). In addition and in line with the above results, RA reduced Sp7 (osterix) expression while simultaneously increasing expression of both Tnfsf11 (RANKL) and Dmp1. We did not find increased Ctsk staining of osteocytes in hypervitaminosis A or increased Ctsk mRNA expression in RA treated osteoblastic cells (Fig 4C). Furthermore, similarly to undifferentiated MC3T3-E1 cells (preosteoblasts), also differentiated MC3T3-E1 cells (mature osteoblasts or osteocytes) responded to RA treatment with a rapid increase in the expression of Dmp1, RANKL, Acp5, Mmp9 and Mmp13 (Fig 4D). Finally, we show that Dmp1 expression is rapidly induced by RA also in primary mouse calvarial osteoblasts (Fig 4E). The marker genes for mature osteoblasts or osteocytes, Bglap2 and Ibsp, were rapidly reduced by RA treatment whereas Runx2 mRNA expression was unaffected.

## Discussion

We have shown that an excessive dietary of intake of vitamin A causes a rapid calvarial thinning in mice, and that the effects involve all three types of bone cells: osteoblasts, osteoclasts and osteocytes. First, and in accordance with our previous study demonstrating that vitamin A is a negative regulator of osteoblast mineralization [6], we could show that excess vitamin A reduced the bone formation rate on the pericranial side (the major bone-forming site in the skull) and the calvarial bone density. Notably, we did not see any signs of craniosynostosis, a feature of developmental exposure to high vitamin A levels, in these 8-week old mice. In contrast, we found suture diastasis and bone hypoplasia in the frontal bone plates. These findings are consistent with human cases of postnatal hypervitaminosis A with calvarial decalcification/thinning and suture diastasis [15,20,21].

Second, and in line with vitamin A being a well-known bone resorption stimulator, we found a >6-fold increase in the osteoclast number in the calvaria of hypervitaminosis A mice. Remarkably, and in contrast to other resorption stimulators such as interleukin-1 beta (IL-1 beta), tumor necrosis factor alpha (TNF-alpha), parathyroid hormone (PTH), parathyroid hormone-like protein (PTHrP) and 1,25-dihydroxyvitamin D (1,25(OH)_2_D_3_), vitamin A induced endocranial rather than intracranial osteoclastogenesis in calvarial bone [22]. Together with the observations by Barnicot and Datta of vitamin A-generated holes in the scapula and mandible [7], the finding in our present study strongly suggests that the calvarial perforations found in humans, mice and zebrafish lacking CYP26B1 are, at least partly, explained by increased osteoclastic bone resorption at the endocranial bone surface. A recent study in zebrafish further strengthens the connection between calvarial perforations, osteoclasts and vitamin A [23].

The early histological studies by Barnicot and Datta also describe that blood vessels are enlarged or engorged at locations close to increased osteoclastogenesis in animals with hypervitaminosis A [7]. Using immunohistochemistry we could confirm their observations, both in this study and in an earlier study of rat long bones [19]. It is well known that osteoclasts and their precursors originate from hematopoietic cells of the monocyte/macrophage linage present in the bone marrow and peripheral circulation, and that osteoclasts develop and function in close proximity to the bone microvasculature and sinusoids. In fact, angiogenesis and osteoclast formation is increasingly recognized to be linked [24–26]. Here we found enlarged blood vessels in the well perfused dura mater membrane covering the endocranial bone surface in hypervitaminosis A. In addition, we observed increased staining of Icam1, a key endothelial molecule involved in active recruitment of osteoclast precursors [25], in the dura mater close to the endocranial osteoclasts found in hypervitaminosis A animals. Importantly, these results are in agreement with our previous study where we found that osteoclastogenesis was intimately associated not only with blood vessel Icam1 expression, but also altered blood flow around the cortical bone [19,27]. Taken together, we may speculate that vitamin A induces Icam1 expression on blood vessels close to the endocranial bone, followed by increased endothelial adhesion and transendothelial migration of osteoclast precursors. These osteoclast precursors are then brought in contact with RANKL-expressing osteoblasts/osteocytes which activate them to become mature osteoclasts. Along these lines we have previously shown [6] that RA induces accumulation of the scarce full-length RANKL protein, the key molecule for osteoclastogenesis, in osteoblastic cells [28,29]. *In vivo*, both bone and osteoblasts/osteocytes are necessary for osteoclastogenesis [30–33]. Similarly to the *in vivo* situation, RA induces osteoclastogenesis in bone organ cultures, which represent essentially intact bone tissue containing both osteoblasts and (pre)osteoclasts [8–11]. In contrast, RA inhibits osteoclastogenesis in pure (pre)osteoclastic cultures [34,35] although it can moderately increase bone-resorbing activity of mature osteoclasts [36–38]. Thus, it seems likely that vitamin A induces osteoclastogenesis indirectly via increased osteoblastic/osteocytic RANKL expression. In fact, we show here that RA rapidly induces Dmp1 and RANKL expression in both preosteoblastic and osteoblastic/osteocytic cells. Moreover, we demonstrate that also primary osteoblasts rapidly respond to RA with increased Dmp1 expression, as we earlier have shown for RANKL expression [39]. These *in vitro* findings corroborate our *in vivo* observation (using immunohistochemistry) of increased Dmp1 staining intensity of endocranial osteocytes in immediate contact with large osteoclasts, situated in deep resorption pits. Together this suggests that the Dmp1 positive endocranial osteocytes in the vitamin A-fed mice also express RANKL. Thus, the mechanism by how vitamin A specifically induces endocranial osteoclasts could be via a locally restricted effect on osteocytes close to the endocranial bone surface. That the endocranial osteocytes appear to be the major target of excess vitamin A probably relies on the fact that the endocranial surface is best vascularized. Notably, in our previous study of hypervitaminosis A in rat long bone we found no correlation between RANKL mRNA expression in bone and circulating truncated forms of RANKL protein [19]. This together with the previous observation that the membrane associated full-length protein of RANKL is the key inducer of osteoclastogenesis *in vivo*, motivated us to try to detect the full-length RANKL protein in bone sections with an antibody that worked in Western blotting [6]. Although we were unable to detect the full-length RANKL protein in these sections, we have previously seen a clear co-accumulation of Dmp1 and the full-length RANKL proteins in cultures of RA-treated osteoblasts/osteocytes [6]. These two proteins appear simultaneously after 21 days in culture, suggesting that endocranial Dmp1-positive osteocytes also express low but significant levels of RANKL in the hypervitaminosis A calvaria. This may also be supported by the recent observation that RANKL is associated with TRAP in the same intracellular vacuoles in osteocytes [40]. Accordingly, the osteoclastogenesis induced by hypervitaminosis A in mouse calvaria is not only associated with endothelial Icam1 staining but also with osteocytic Dmp1 staining, similarly to what we have seen in rat long bones [6,19]. We also noticed that the noncollagenous bone matrix genes Bglap2 and Ibsp, mostly expressed by mature osteoblasts/osteocytes, were rapidly reduced by RA. Along this line we have shown earlier that long term RA treatment of MC3T3-E1 cells suppress Phex, Sost and Fgf23 expression [6].

Osteocytes, the most abundant cells in bone, have recently been recognized to play a significant role in mineral homeostasis [41]. Both PTHrP and vitamin D can induce osteocytes to demineralize their perilacunar area to release calcium (and phosphate) [42,43]. In particular, PTHrP infusion in mice was shown to increase both TRAP and Ctsk expression in osteocytes [43]. Osteocytic osteolysis has previously been suggested to occur in hypervitaminosis A, based on histological examinations [44,45] and an increase in osteocyte lacuna size has been reported in man after high intake of vitamin A [46]. Here, we add further support for such osteocytic osteolysis with the observation that osteocytes express TRAP and have increased non-bone matrix staining in their immediate surroundings, similarly to what has been observed for PTHrP-induced osteocyte bone remodeling [43]. That the osteocytic TRAP-staining was indeed a vitamin A effect was corroborated by the finding that RA increased TRAP/Acp5 expression in osteoblastic cells. Although we did not observe increased Ctsk expression by RA in osteoblastic cells, which has been observed after PTHrP treatment [43], we found that RA induced osteoblastic expression of collagen degrading Mmp9 and Mmp13. In fact, similarly to PTH, 1,25(OH)_2_D_3,_ and prostaglandin E_2_, RA rapidly induces osteoblastic collagenase expression and collagen breakdown in bone organ cultures [8,47,48]. Importantly, and in line with that vitamin A induces osteoclastogenesis indirectly via osteoblasts, calvarial bone organ cultures have been used to show that RA has immediate effects on osteoblast markers (hours) whereas osteoclast changes take days [10,11]. Thus, taken together with the reduced bone density detected by μCT measurements, our results indicate that vitamin A reduces calvarial bone mineral density also by directly inducing osteocytic osteolysis, via a rapid increase in expression of bone degrading genes in osteocytes.

In conclusion, we have shown that a high dietary intake of vitamin A rapidly induces thinning of the calvaria in mice by reducing pericranial bone growth and mineralization, increasing bone resorption and by osteocytic osteolysis. Vitamin A appears to be unique among the agents known to induce bone resorption by predominantly increasing resorption on the endocranial side of the bone. Clinical cases of hypervitaminosis A are still seen in both infants [49] and adults [50]. Our present findings together with previous studies indicate that examination of the calvaria may give valuable information in these cases.

## Materials and methods

### Animals and experimental design

This study was carried out in strict accordance with the recommendations in the Guide for the Care and Use of Laboratory Animals of Sweden. The protocol was approved by the Committee on the Ethics of Animal Experiments of the University of Uppsala (Permit Number: C 275/12) and the methods were carried out in accordance with the approved guidelines. Twenty female C57BL6 mice were obtained from Möllegaards Breeding Centre, Ltd. (Skensved, Denmark). They were acclimatized for one week and kept in groups of five animals and had free access to water. The mice were divided into 2 experimental groups with 10 mice in each. They were fed a standard diet (Lactamin R36, Stockholm, Sweden) containing 12 IU vitamin A/g pellet (“Control”), or a standard diet supplemented with 1700 IU vitamin A/g pellet (“Vitamin A”). This was the same diet as we used in our previous study on rats [6], where total vitamin A serum levels increased 93% after 7 days of ingestion. All animals received a calcein injection at day 0 and at day 5. The vitamin A was added to the pellets in the form of retinyl palmitate and retinyl acetate. At the end of the experiment (day 7), the mice were sacrificed by cervical dislocation under sodium pentobarbital anesthesia.

### Peripheral quantitative computed tomography (pQCT)

The left femur (n = 10/group) was scanned using pQCT (Stratec XCT Research SA+) with version 5.50 R software (Norland Stratec Medizintechnik, Pforzheim, Germany) using a voxel size of 0.07 mm and a scan speed of 3mm/sec. For the cortical parameters we analyzed a single mid-diaphyseal section of the femur using the CORTBD algorithm with the cortical threshold set at 400 mg/cm^3^.

### Micro-computed tomography (μCT)

The bone morphology was evaluated by means of micro-computed tomography (Skyscan 1072, Bruker, Kontich, Belgium). Specimens were acquired using: source voltage: 60 kV; current: 167 μA; filter: 0.5 mm aluminium; and an isotropic pixel size of 5 μm^2^. Reconstruction of cross sections was done using software package NRecon (SkyScan, Bruker, Kontich, Belgium), which uses a filtered back-projection algorithm. Three-dimensional reconstructions of the samples were obtained using CTvox (SkyScan, Bruker, Kontich, Belgium).

### Histomorphometry

This analysis was conducted by Pharmatest Services Ltd, Turku Finland. Histomorphometry was performed to analyze mineralized bone area, void area, osteocyte density, osteoclast number and surface, mineralizing surface, mineral apposition rate, and bone formation rate in undecalcified mouse calvaria [51,52]. Each sample (n = 8) was dehydrated in an increasing series of EtOH concentrations, defatted in xylene, and embedded in methyl methacrylate. The embedded sample was sectioned in a coronal plane using a fully automated rotary microtome (Leica RM2265; Leica Microsystems, Wetzlar, Germany) and a tungsten-carbide knife. Plastic sections with the thickness of 4 μm were obtained from parietal bones at the site dorsal from the intersection of coronal and sagittal sutures. Three sections from each sample were stained in von Kossa and the area of the entire bone, stained bone and voids were determined in one representative section. Three sections from each sample were stained also in Masson-Goldner's Trichrome (MGT) and the number of osteocytes and osteoclasts as well as the osteoclast surface were analyzed in one representative section. The analysis of von Kossa and MGT-stained sections were performed in 3-mm-long regions in both parietal bones. Osteoclast parameters were analyzed separately on the pericranial, endocranial and intracranial surfaces of these regions. In addition, three unstained sections were obtained from each sample and analyses of mineralizing surface, mineral apposition rate and bone formation rate were performed in one representative unstained section. These dynamic parameters were analyzed on the pericranial, endocranial and intracranial surfaces of both parietal bones, and reported for each parietal bone separately and for their combination. All analyses were performed using an automated upright microscope system (Leica DM4000 B; Leica Microsystems) and a microscopy automation & image analysis software (MetaMorph; Molecular Devices, Sunnyvale, CA, USA).

### Immunohistochemistry

The skin was removed from the skull and divided in two halves by a coronal cut followed by immersion-fixation for 48 h in 10% phosphate buffered formaldehyde, decalcified in a Sakura TDE^™^ 30 decalcifier system, dehydrated and embedded in paraffin wax. Sections (5 μm thickness) were transferred to slides (SuperFrost, Menzel-Gläser, Germany) and then deparaffinized and rehydrated. The sections were treated with H_2_O_2_ to block endogenous peroxidase activity and with trypsin for antigen retrieval (Digest-All 2, Life Technologies). (Trichrome Stain, Masson, Kit, Sigma-Aldrich) and Tartrate resistant acid phosphatase (TRAP) staining (Acid Phosphatase Leukocyte staining kit, Sigma-Aldrich). Immunostaining for cathepsin K was achieved by the use of a polyclonal anti-mouse cathepsin K antiserum at a dilution of 1:300 [53], Dmp1 1:400 rabbit polyclonal anti-Dmp1 (M176) (Takara Bio Inc., Japan) and goat polyclonal anti-Pecam1 and anti-Icam1/CD54 (1:500, R&D Systems). Counterstaining was performed with Ehrlich’s hematoxylin. Visualization of the antibodies was achieved by incubation with a secondary biotinylated antibody at a dilution of 1:200 in 2% serum and PBS followed by an avidin–biotin– peroxidase complex incubation using the Vectastain ABC-kit (Vector Laboratories) and the substrate diaminobenzidine tetrahydrochloride (DAB, DAKO). Quantification of blue pixels was done in ImageJ.

### Cell culture

The mouse preosteoblast cell line, MC3T3-E1 subclone 4 (from ATCC) was cultured and treated essentially as described before [6]. In brief, control media consists of α-MEM supplemented with 10% heat inactivated fetal bovine serum, 2 mM L-glutamine, 100 μg/ml streptomycin and 100U/ml penicillin. To induce a mature osteoblastic/osteocytic phenotype, confluent cells were treated 8 days with media containing 25 μg/ml ascorbic acid and 10 mM beta-glycerophosphate. Change of media was done every 2nd or 3rd day. Primary mouse osteoblasts were isolated from calvariae of 3-day-old neonates as described before [54]. Briefly, calvariae from four mice were dissected out and incubated in 10 mg/ml collagenase (Type IA, Sigma; in HBSS) for 15 min at 37°C with shaking. The supernatant was discarded and the calvariae transferred into fresh collagenase and incubated as before for 30 min. The cells were collected by centrifugation, resuspended in HBSS and stored on ice. The calvariae were then incubated with 4 mM EDTA for 15 min, again the cells were pelleted, resuspended and placed on ice. The calvariae were further treated with collagenase for 30 min and the three fractions were pooled and plated in a 75-cm^2^ flask. Undifferentiated MC3T3-E1 and the primary osteoblasts were seeded 24h prior to addition of control medium with or without 400 nM RA for another 24h. The mature osteoblastic/osteocytic MC3T3-E1 cells received differentiation media with or without 400 nM RA at day 8. RA was dissolved in dimethyl sulfoxide (DMSO). At the end of the experiment total RNA was extracted using TRI Reagent^®^ (Sigma-Aldrich). Each experiment was performed at least three times using triplicates.

### Quantitative RT-PCR

Four hundred ng of total RNA was transcribed to cDNA using the TaqMan system (Applied Biosystems, USA). Quantitative real time PCR was performed using inventoried TaqMan^®^ Gene Expression Assays for *Runx2* ENSMUSG00000039153 (Mm00501584_m1), *Acp5 (Trap)* ENSMUSG00000001348, (Mm00475698_m1), *Sp7 (Osterix)* ENSMUSG00000060284 (Mm00504574_m1), *Dmp1* ENSMUSG00000029307 (Mm01208363_m1), *Tnfsf11 (RANKL)* ENSMUSG00000022015 (Mm00441908_m1), *Ctsk* ENSMUSG00000028111 (Mm00484036_m1), *Mmp9* ENSMUSG00000017737 (Mm00442991_m1), *Mmp13* ENSMUSG00000050578 (Mm00439491_m1), Ibsp ENSMUSG00000029306 (Mm00492555_m1) and *Bglap2 (osteocalcin)* ENSMUSG00000074486 (Mm03413826_mH), according to the manufacturer's protocol, on a TaqMan 7000 apparatus. Cycling protocol: 50°C for 2 min, followed by 95°C for 10 min and then 40 cycles of 95°C 15 sec followed by 60°C for 1 min. For standardization, expression levels were divided by expression level for *Actb* ENSMUSG00000029580 (Mm00607939_s1), derived from dilution standard curves of Ct values for each gene. Each experiment was performed at least three times using triplicates.

### Statistical analyses

The Student’s t-test was used for all analyses. In every case, p<0.05 was considered statistically significant.

## Supporting information

S1 Fig Endo- and intracranial bone and mineral apposition rates. (DOCX)
