# Supplementary figures and images for: Excessive dietary intake of vitamin A reduces skull bone thickness in mice
Source: PLoS One. 2017 Apr 20;12(4):e0176217. doi: 10.1371/journal.pone.0176217 (PMC5398668; doi:10.1371/journal.pone.0176217)

S1 Fig


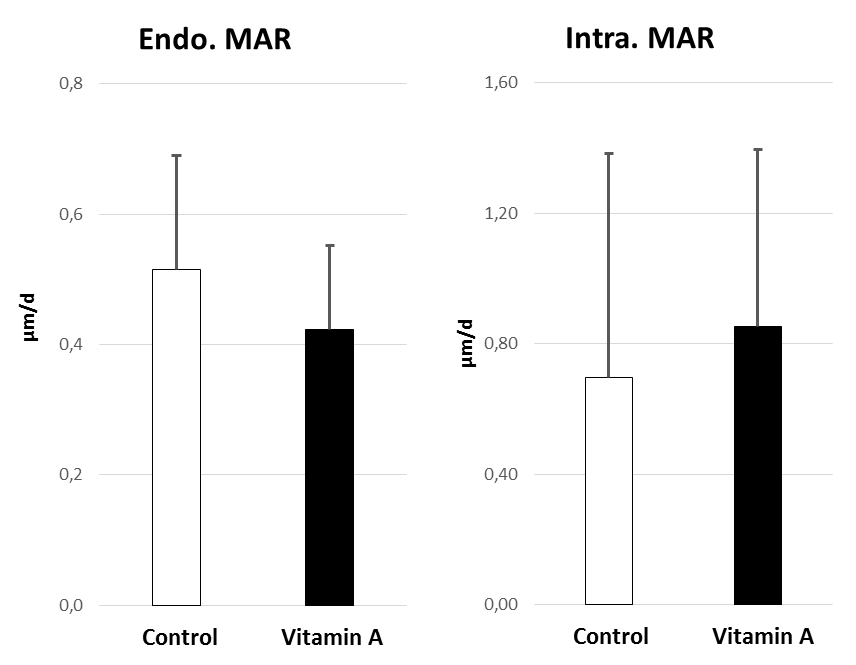


Endo- and intracranial bone and mineral apposition rates. Results are given as means + SD.

Supplement: S1 Fig — (DOCX) [file pone.0176217.s001.docx]
